# Cost comparison of laparoscopic versus open surgery for common procedures in Rwandan teaching hospitals

**DOI:** 10.1016/j.sopen.2025.07.001

**Published:** 2025-07-08

**Authors:** King Kayondo, Martin Nyundo, Miguel Gasakure, Janvière Mutamuliza, Leon Ngeruka, Regis Hitimana, Julien Gashegu, Annie Robert

**Affiliations:** a Department of Surgery, University of Rwanda, Kigali, Rwanda; b Department of Surgery, University Teaching Hospital of Kigali, University of Rwanda, Rwanda; c Rwanda Military Referal and Teaching Hospital, Rwanda; d Rwanda Social Security Board, Rwanda; e Clinical Anatomy Department, University of Rwanda, Rwanda; f Epidemiology & Biostatistics, Institut de Recherche Expérimentale et Clinique, Université Catholique de Louvain, Belgium

**Keywords:** Laparoscopy, Open surgery, Cost comparison, Healthcare costs, Clinical outcome, Healthcare accessibility, Health insurance

## Abstract

**Objective:**

This study evaluates the economic and clinical impacts of minimally invasive surgery (MIS) compared to open surgery (Open S) for four common procedures—appendectomy, cholecystectomy, hernia repair, and ovarian cystectomy—at two major teaching hospitals in Rwanda, RMRTH and CHUK. The aim is to assess direct costs, hospital stays, complications, and recovery times for MIS versus Open S and to explore the role of health insurance in MIS accessibility.

**Methods:**

A retrospective analysis was conducted on data from 206 patients treated between 2019 and 2022, with 100 undergoing Open S and 106 receiving MIS. Data included direct costs, hospital stay lengths, post-operative complications, and recovery times. The study also examined the correlation between MIS utilization and health insurance.

**Results:**

The average patient age was 41.7 years, with nearly equal gender distribution (52.4 % male, 47.6 % female). Most patients (79.1 %) had Community-Based Health Insurance coverage. Laparoscopic cholecystectomy showed significant economic advantages, with shorter stays, fewer complications, and faster recovery (*p* < 0.02). MIS for hernia repair offered quicker recovery but incurred higher costs. For appendectomy and ovarian cystectomy, there was no significant cost difference between MIS and Open S. A strong positive correlation was found between MIS adoption rates and health insurance, supporting improved access.

**Conclusion:**

MIS in Rwanda shows promise for economic savings, better patient outcomes, and expanded access through insurance. However, challenges like high consumable costs and limited expertise need to be addressed to fully optimize MIS benefits in Rwanda's healthcare system.

## Introduction

Surgical interventions are essential to addressing global health needs, yet access to safe and affordable surgical services remains a critical challenge, particularly in developing countries. Despite advancements in surgical technologies, the burden of surgical disease remains substantial in Developing countries, impacting individuals, families, and entire communities. According to the Lancet Commission on Global Surgery, approximately 5 billion people worldwide lack access to safe, affordable surgical and anesthesia care, with 9 out of 10 of those affected living in Developing countries [1]. The global surgical care gap leads to approximately 18.6 million deaths annually, with untreated surgical conditions accounting for 32.9 % of the global burden of disease [2]. Additionally, Developing countries face significant economic consequences due to untreated surgical conditions, with an estimated loss of up to 2 % of gross domestic product (GDP) annually [2].

Minimally invasive surgery (MIS), particularly laparoscopic surgery (Lap S), offers several advantages over traditional open surgery (Open S). These include reduced postoperative pain, faster recovery, shorter hospital stays, and fewer postoperative complications, all of which contribute to overall cost savings and improved patient outcomes [[3], [4], [5]]. Despite these benefits, the adoption of MIS has been slow in Developing countries due to the high cost of equipment, lack of trained personnel, and financial barriers for patients [[6], [7], [8]]. Moreover, while the upfront costs of MIS may appear higher [9,10], studies from high-income countries indicate that total healthcare costs, including postoperative care and recovery, are often lower than for Open S due to reduced hospital stays and fewer complications.

In Rwanda, significant progress has been made in rebuilding the healthcare system following the 1994 Genocide Against the Tutsi, which left the country's health infrastructure in ruins [11,12]. The introduction of Community-Based Health Insurance (CBHI), commonly known as “Mutuelle de Santé”, has improved access to healthcare services, including surgery, for the majority of the population [13]. Additional public insurance schemes, such as the Rwanda Social Security Board (RSSB) for civil servants and Military Medical Insurance (MMI) for security personnel, along with various private insurance providers, further broaden this healthcare coverage.

Over the past decade, Rwanda has made strides in incorporating MIS techniques into hospital practices, thanks to institutional reforms, international partnerships, and capacity-building initiatives [6,14,15]. However, the cost implications of laparoscopic versus Open S in Rwanda remain under-explored, particularly considering the country's limited resources.

This study hypothesizes that laparoscopic surgery is more **economically advantageous** than open surgery for common procedures in Rwanda when considering both direct surgical costs and postoperative care. This hypothesis is grounded in the assumption that, despite higher initial costs, Rwanda's ongoing healthcare infrastructure development and innovations in surgical practice position laparoscopic surgery as a financially viable approach. By potentially reducing overall healthcare expenditures through shorter hospital stays, faster recovery times, and fewer complications, laparoscopy may offer long-term economic benefits.

However, evidence specific to Developing countries, particularly Rwanda, remains scarce. This study aims to bridge this gap by conducting a comparative cost analysis of laparoscopic and open surgery for common procedures in two major Rwandan teaching hospitals. Given Rwanda's ambition to achieve universal health coverage (UHC) by 2030, a comprehensive understanding of the economic efficiency of different surgical techniques is crucial for optimizing resource allocation and improving healthcare outcomes [16,17].

Globally, studies have highlighted the long-term cost-effectiveness of MIS, yet evidence specific to Developing countries, especially Rwanda, is scarce [9]. With Rwanda's goal to achieve universal health coverage (UHC) by 2030, understanding the cost dynamics of different surgical approaches is essential for optimizing resource allocation and improving healthcare outcomes [16,17].

This study aims to compare the costs of laparoscopic versus Open S for common procedures in two leading Rwandan teaching hospitals. By assessing the cost comparison, the study's findings seek to inform policy decisions, optimize surgical resource allocation, and improve healthcare accessibility in Rwanda.

## Material and methods

### Study design and setting

This retrospective cross-sectional study was conducted at two major teaching hospitals in Rwanda: the University Teaching Hospital of Kigali (Centre Hospitalier Universitaire de Kigali:CHUK) and Rwanda Military Referral and Teaching Hospital (RMRTH). These hospitals were selected due to their diverse patient population and their capacity to handle a wide range of surgical cases. The study period encompassed four years, from January 1, 2019, to December 31, 2022, and included all consecutive patients who underwent either laparoscopic or open surgery for common conditions such as **appendectomy**, cholecystectomy, hernia repair, and ovarian cystectomy.

### Study population and inclusion criteria

The study included patients aged 15 years and above who underwent surgery for one of the targeted conditions at CHUK or RMRTH during the study period. Patients were eligible for inclusion if they had complete medical records, including detailed cost information. Exclusion criteria included patients with incomplete data or those who underwent procedures other than the four targeted conditions. During the study period, based on available hospital data and prior reports, we estimate that approximately 207 laparoscopic and 520 open surgeries were performed in total during this period. While this is only an approximation, our sample was systematically selected and is representative of the surgical caseload at both institutions. After applying the inclusion and exclusion criteria, 106 laparoscopic and 100 open surgery cases were included for the final analysis.

### Data collection

Data were systematically collected from multiple sources, including outpatient consultation registers, operating room logs, hospitalization ward records, and the Open Clinic patient management system (OpenClinic of The Open-IT Group Ltd., Open-IT Rwanda).

Two teams of data collectors trained for consistency and accuracy captured data on patient demographics (age, sex, insurance status), surgical details, pathology, investigations, anesthesia, postoperative complications, medications, and financial information related to the cost of surgery and postoperative care. Cost data included direct costs of surgery (surgical consumables, anesthesia, medications), hospitalization costs, and costs related to the management of postoperative complications. Financial records were verified by hospital cashiers to ensure accuracy.

### Data analysis

Data was recorded using Microsoft Excel and later imported into Stata version 13 (College Station, Texas 77,845 USA) for analysis. Descriptive statistics were used to summarize demographic and clinical characteristics, with categorical data presented as frequencies and percentages, while continuous data were summarized using medians with Minimum and Maximum range. A nonparametric Mann-Whitney *U* test was employed to compare cost differences between laparoscopic and Open S groups, given the skewed distribution of cost data. Statistical significance was defined as *p* < 0.05.

Postoperative complications were classified using the Clavien-Dindo Classification (CDC) system [18,19], allowing for a standardized comparison of complications between the two surgical approaches. The analysis focused on both the occurrence and severity of postoperative complications, which were then used to assess any impact on the total cost of care.

### Ethical considerations

Ethical approval for the study was obtained from the Rwanda National Ethical Committee (No. 145/RNEC/2023) as well as from the respective ethical committees at CHUK and RMRTH. All patient data were anonymized to protect confidentiality, and access to sensitive information was restricted to authorized personnel. The study adhered to the principles of the Declaration of Helsinki regarding research ethics.

## Results

Table 1 presents the demographic and clinical characteristics of the 206 patients in the study. The MIS group had a significantly higher mean age (48.1 years). Males predominated in the Open S group (73.2 %), while females were more common in the laparoscopic group (78.6 %). Both groups had a similar distribution between CHUK and RMRTH. The most common insurance coverage was CBHI, with 76.4 % in the laparoscopic group and 82.0 % in the Open S group.

**Table 1:** Demographic and clinical characteristics of patients undergoing laparoscopic and open surgery.

Characteristics	Open Surgery	Laparoscopic Surgery
Age (Mean ± SD)	35.1 (±16.5)	48.1 (±15.9)
Sex (n, %)		
Male	79 (73.2 %)	29 (26.8 %)
Female	21 (21.4 %)	77 (78.6 %)
Hospital of Recruitment -no. (%)		
CHUK*	44 (48.9 %)	46 (51.1 %)
RMRTH**	56 (48.3 %)	60 (51.7 %)
Insurance Coverage -no. (%)		
CBHI^$^	82 (82.0 %)	81 (76.4 %)
MMI ^$$^	12 (12.0 %)	11 (10.4 %)
RSSB^$$$^	5 (5.0 %)	6 (5.7 %)
Private Insurance	1 (1.0 %)	7 (6.6 %)
No Insurance	0 (0.0 %)	1 (1.0 %)

CHUK*: Centre Hospitalier Universitaire de Kigali, RMRTH**: Rwanda Military Referral and Teaching Hospital.

**CBHI**^$^: Community Based Health Insurance, **MMI**^$$^: Military Medical Insurance,

**RSSB**^$$$^: Rwanda Socio Security Board.

Table 2 shows the distribution of surgical procedures by approach. Laparoscopic cholecystectomy was the most common (90.2 %), while open surgery was preferred for hernia repairs (85 %) and appendectomies (75.9 %). Laparoscopy was the dominant approach for ovarian cystectomy (86.7 %).

**Table 2:** Surgical procedures by surgical approach.

Surgical Procedure	N	Open Surgery n (%)	Laparoscopic Surgery n (%)
Cholecystectomy	82	8 (9.8 %)	74 (90.2 %)
Hernia Repair	80	68 (85 %)	12 (15 %)
Appendectomy	29	22 (75.9 %)	7 (24.1 %)
Ovarian Cystectomy	15	2 (13.3 %)	13 (86.7 %)

Table 3 shows the distribution of surgical procedures by gender. Hernia repairs were predominantly performed on males (83.8 %), while cholecystectomy was more common among females, especially in the MIS group (80.5 %).

**Table 3:** Surgical procedures by gender.

Procedure	N	Male n (%)	Female n (%)
Cholecystectomy	82	16 (19.5 %)	66 (80.5 %)
Hernia Repair	80	67 (83.8 %)	13 (16.2 %)
Appendectomy	29	25 (86.3 %)	4 (13.7 %)
Ovarian Cystectomy	15	0 (0.0 %)	15 (100.0 %)

Table 4 summarizes complications in open surgery (Open S) and laparoscopic surgery (Lap S) based on the Clavien-Dindo classification. Open S had more complications, particularly in cholecystectomy, including acute liver injury, bile duct injury, and peritonitis (Grade 3B). Lap S reported one major complication—a pulmonary embolism during cholecystectomy (Grade 4). No complications were observed in Lap S appendectomy.

**Table 4:** Clinical outcomes by procedure and postoperative complications by Clavien-Dindo classification.

Surgery Type	Open Surgery			Laparoscopic Surgery		
	Complication	N	Grade	Complication	N	Grade
Cholecystectomy						
	Acute liver injury	1	2	Pulmonary embolism	1	4
	Bile leak/bile duct injury	1	2			
	Peritonitis	1	2			
	Subhepatic collection	1	2			
Appendectomy						
	Peritonitis	1	3B	None		
	Surgical site infection	1	2			
Hernia Repair						
	Intestinal obstruction	1	2	None		
Ovarian Cystectomy						
	None			None		

Table 5 compares hospital stay duration between open surgery (Open S) and laparoscopic surgery (Lap S). Lap cholecystectomy had a significantly shorter stay (median 3 days) than Open S (median 8 days, p < 0.001). Similarly, ovarian cystectomy patients had a shorter stay with MIS (median 2 days) vs. Open S (4 days), p = 0.001. No significant difference was observed for hernia repair and appendectomy between the two approaches.

**Table 5:** Length of hospital stay by procedure.

Surgery Type	Open Surgery (n)	Laparoscopic Surgery(n)	Median, Hospital Stay, N:(Min-Max)	*P*-value
Cholecystectomy	8	74	OpenS*: 8 (4–30), LS**: 3 (1–39)	<0.001
Hernia Repair	68	12	OpenS: 3 (0−10), LS: 3 (2–5)	0.837
Appendectomy	22	7	OpenS: 4 (1–19), LS: 3 (2–5)	0.353
Ovarian Cystectomy	2	13	OpenS: 4 (4–4), LS: 2 (2–3)	0.001

OpenS*: Open Surgery, LS**: Laparoscopic Surgery.

Table 6 compares median medical costs for cholecystectomy, hernia repair, appendectomy, and ovarian cystectomy between laparoscopic surgery (Lap S) and open surgery (Open S), highlighting significant cost differences in some cases.

• Cholecystectomy: Lap S (133,433 RWF) was significantly cheaper than Open S (240,461 RWF) (*p* = 0.016).
• Hernia repair: Lap S (220,752 RWF) was costlier than Open S (119,296 RWF) (*p* = 0.001), favoring open surgery.
• Appendectomy: Lap S (242,895 RWF) vs. Open S (195,432 RWF) (*p* = 0.838) showed no significant cost difference.
• Ovarian cystectomy: Lap S (103,079 RWF) vs. Open S (136,868 RWF) (*p* = 0.671) also showed no significant cost difference.

**Table 6:** Medical cost in Rwandan Francs (RWF*).

Surgical Technique	Cholecystectomy Median (Min-Max)	Hernia repair Median (Min-Max)	Appendectomy Median (Min-Max)	Ovarian cystectomy Median (Min-Max)
Lap S	133,433 (46,693-2,426,040)	220,752 (178,728-525,946)	242,895 (74,383-277,182)	103,079 (74,836-215,754)
Open S	240,461 (97,220-643,116)	119,296 (54,935-383,375)	195,432 (61,962–410.032)	136,868 (73,778-199,959)
*P* value	0.016	0.001	0.838	0.671

RWF*: Rwandan Franc.

Overall, Lap S offers cost savings for some procedures, but hernia repair remains more economical with Open S.

Both hospitals follow similar national procurement policies, so there are no major cost differences and we did not conduct a detailed cost breakdown by hospital.

Table 7 details cost drivers for laparoscopic (Lap S) and open surgeries (Open S) across key categories: surgical acts, anesthesia, nursing, consumables, medications, and hospitalization.

• Out-of-pocket (OOP) costs are generally higher for Lap S**.**
• Surgical acts**:** Lap S (49,116.21 RWF) vs. Open S (22,653.18 RWF)
• Anesthesia & nursing**:** Higher for Lap S
• Consumable costs are greater for Open S, while medication costs are higher for Lap S**.**
• Cost differences stem from consumable volume, hospital stay, medication type (IV vs. oral), and anesthesia methods**.**
• Insurance expenses are higher for Open S, suggesting coverage influences patient costs differently across techniques**.**

**Table 7:** Cost drivers.

Area	Open Surgery (RWF⁎⁎)				Laparoscopic Surgery (RWF⁎⁎)			
	OOP⁎	Insurance	Total	*Tot Euro*	OOP	Insurance	Total	*Tot Euro*
Surgical Act	7496	21,980	29,476	*26.0*	13,252	28,373	41,625	*36.7*
Anesthesia acts	1611	14,501	16,112	*14.2*	2225	18,075	20,300	*17.9*
Nursing acts	2723	24,286	27,009	*23.8*	7009	19,060	26,069	*23.0*
Consumables	5409	48,678	54,087	*47.7*	6209	40,689	46,898	*41.3*
Drugs/medi- cations	3622	32,596	36,218	*31.9*	14,015	39,533	53,548	*47.2*
Hospitalization	1793	12,000	13,793	*12.2*	6406	13,577	19,983	*17.6*
Total	22,654	154,041	176,695	*155.7*	49,116	159,307	208,423	*183.7*
*Tot Euro*	*20.0*	*135.7*	*155.7*		*43.3*	*140.4*	*183.7*	

1 RWF = 0.0008812 Euro (26 Dec 2022, Ref: Exchange-rates.org).

⁎ Out of pocket.

⁎⁎ Rwandan Franc.

Overall, Lap S incurs higher OOP costs, while insurance covers more in Open S.

Here, OOP expenses refer specifically to the amount paid directly by the patient, as recorded in the hospital's accounting system, and not covered by insurance. This includes co-payments and any other payments made within the hospital system for procedures, medications, consumables, or other healthcare services.

Table 8 summarizes the cost-benefit comparison between open surgery (Open S) and laparoscopic surgery (Lap S) across various procedures. This analysis highlights potential cost savings or increases associated with each surgical method.

**Table 8:** Cost ratio.

Procedure	Mean stay	Mean cost per procedure (RWF⁎)	Cost per mean stay (RWF⁎)	Cost-Benefit Ratio (%)
Cholecystectomy Open S	11.3	347,453	50,551.6	+34.9
Lap S	4.2	226,119	159,146.4	
Hernia repair				
Open S	3.0	159,787	94,116.65	−67.5
Lap S	2.9	267,635	159,402.68	
Appendectomy				
Open S	5	199,538	95,172.04	−13.7
Lap S	3.5	226,836	125,149.26	
Ovarian cystectomy				
Open S	3.7	271,081	82,400.34	+40.1
Lap S	3.2	162,318	98,121.68	

⁎ : Rwandan Francs.

Laparoscopic cholecystectomy procedure (226,119 RWF) is 34.9 % less expensive than open surgery (347,453 RWF), and laparoscopic ovarian cystectomy (162,318 RWF) shows a 40.1 % cost reduction compared to its open counterpart (271,081 RWF). Conversely, laparoscopic hernia repair (267,635 RWF) is 67.5 % more expensive than the open technique (159,787 RWF), and laparoscopic appendectomy (226,836 RWF) costs 13.7 % more than open surgery (199,538 RWF).

## Discussion

This study provides valuable insights into the utilization and outcomes of MIS compared to Open S at Rwanda's two largest University Teaching Hospitals, along with their economic implications. Focusing on patient groups undergoing Cholecystectomies, Ovarian cystectomies, Hernia Repairs, and Appendectomies, the research highlights both the advantages and challenges of MIS implementation within the country's healthcare system. The choice of surgery was determined based on the patient's clinical condition, the surgeon's expertise, and the patient's preference. None of the laparoscopic surgeries were converted to open due to clinical or perioperative challenges in our dataset. The findings offer a comprehensive understanding of the economic implications of these surgical approaches, alongside their clinical outcomes. This emphasizes the critical importance of healthcare accessibility, affordability, and equity.

MIS for cholecystectomies emerged as the most financially advantageous procedure among those studied, consistent with findings from other studies [20]. The economic advantages can be attributed to several factors:

Shorter Hospital Stays: Patients undergoing Lap Cholecystectomy have shorter hospital stays compared to those undergoing Open S. This reduction in hospitalization time leads to lower room and board charges, contributing to overall cost savings.

Fewer Complications: The minimally invasive approach is linked with a lower risk of post-operative complications such as infections, bleeding, and wound healing issues. By minimizing these adverse events, MIS reduces the need for additional treatments, medications, and extended hospital stays, thereby lowering post-operative care costs.

Quicker recovery allows patients to return to their normal activities, including work, sooner. This expedited recovery not only enhances patients' quality of life but also decreases indirect costs related to lost productivity and wages.

On the other hand, MIS Hernia repairs often entail higher costs than Open S, a finding supported by several studies [21,22].

However, laparoscopic Hernia repair offers advantages such as fewer complications, shorter hospital stays, reduced post-operative pain, decreased readmissions, fewer outpatient visits, and less time off work compared to open repair [23].

Interestingly, no significant cost difference was found between MIS and open procedures for appendectomies and ovarian cystectomies. This aligns with findings from Nascimento et al. [24] and by Adisa A et al. in Nigeria [25] though differs from the study Vellani Y et al. in Pakistan [26], which indicated a slightly higher overall cost for laparoscopic appendectomy. Further studies have highlighted the cost-effectiveness of laparoscopic appendectomy compared to Open S [27]. Benefits such as reduced societal costs, a lower infection rate, and a quicker return to work remained consistent across studies when comparing laparoscopic to open appendectomy and ovarian cystectomy.

Cholecystectomy was identified as the most frequently performed procedure in this study, predominantly carried out using MIS techniques. This aligns with global trends, as cholecystectomy is widely regarded as an ideal procedure for MIS due to its accessibility, predictability, cost-effectiveness, and quick recovery, establishing it as the gold standard for gallbladder removal [[28], [29], [30]].

Moreover, Rwanda currently observes a growing trend of MIS surgeries with the advent of MIS formal training as a fellowship program with the support of Belgian universities cooperation through “Académie de Recherche et d'Enseignement Supérieur, ARES” [31,32] with cholecystectomy being a common entry point for beginners. It constitutes the most frequently performed operation in our hospitals, owing to its relatively straightforward operative technique.

On the other hand, hernia repair remained the most commonly performed Open S, indicating that certain procedures still rely primarily on traditional techniques. The variation in Laparoscopic Hernia repair adoption can be attributed to factors such as higher operative costs, the expense of surgical meshes, and a shortage of trained laparoscopic surgeons [22].

While MIS offers societal benefits compared to open procedures, this distinction is especially critical in Developing countries like Rwanda. In these hospital settings, patients often depend on external sources for food, require frequent visits from family members, or need a companion to stay with them. These factors contribute to additional costs, imposing economic burdens on households [25]. Given that 60.5 % of the Rwandan population is aged between 16 and 64 years [33], early hospital discharge is crucial, as patients frequently serve as primary income earners for their households.

Regarding complication rates, the study found varying outcomes between Open S and MIS groups for different procedures. While the Open S group for cholecystectomy experienced several complications, including acute liver injury, bile leak/bile duct injury, peritonitis, and subhepatic collection, the MIS group reported a single case of pulmonary embolism. The patient received treatment and the follow up was uneventful.

Despite promising results, challenges in MIS adoption remain. Appendectomy and hernia repair were still primarily performed as Open S, indicating potential barriers to the wider implementation of MIS for these procedures. Addressing these barriers may involve improving surgical training and resources, raising awareness among healthcare professionals, and educating patients to help them make informed choices.

This study identifies key cost drivers in MIS procedures in Rwanda, namely, pharmaceuticals and consumables. Open S incurs higher consumable costs, while MIS shows higher medication expenses. Factors like consumable type, anesthesia choice, and postoperative care contribute to these cost differences. For instance, prolonged hospital stays in Open S increase consumable usage, while MIS requires general anesthesia, impacting medication expenses. It is also essential to note that surgical procedures, along with consumables and medical devices for both techniques, are standardized across all hospitals according to their level, in compliance with Rwanda's national healthcare price regulation.

Another important point is that some MIS consumables are not recorded in hospital books, primarily because they are unavailable or not covered by insurance. Patients often obtain these items directly from external suppliers, bypassing hospital procurement channels and records, which leads to an underestimation of the cost of MIS consumables in the study.

Consumables encompass a broad spectrum of disposable medical supplies, surgical instruments, implants, and other materials indispensable for performing MIS procedures. The cost of these consumables can vary based on their type, quality, brand, and supplier. Additionally, due to the rarity of certain cases, specific consumables may not be readily available. Surgeons sometimes face the dilemma of proceeding with an Open S or postponing until the required consumables are accessible.

Hospitalization costs are significantly influenced by the duration of hospital stay required for each MIS procedure. Reducing hospitalization duration through efficient preoperative planning, surgical techniques, postoperative care, and patient management can result in substantial cost savings. Within the context of Rwanda's healthcare system, these costs can vary based on the category of room chosen by the patient, ranging from private rooms to standard accommodations, each with its corresponding cost.

Effective postoperative care strategies, patient education, and transitional care practices can contribute to improved outcomes and reduced long-term costs.

Furthermore, the decision-making process regarding surgical approaches should consider patient-specific factors, including comorbidities, preferences, and potential benefits in terms of recovery time and quality of life. Moreover, healthcare policymakers and providers should weigh the economic considerations alongside clinical effectiveness to optimize resource allocation and patient outcomes.

The study underscores the importance of understanding and managing the key cost drivers associated with pharmaceuticals, and consumables in MIS procedures in Rwanda. Embracing a systematic approach to cost management, incorporating evidence-based practices, standardizing protocols, optimizing resource utilization, enhancing supply chain efficiency, and implementing quality and safety measures are essential to controlling costs, improving value, and delivering high-quality care.

When evaluating Cost ratios across procedures like cholecystectomy, Hernia repair, appendectomy, and ovarian cystectomy, MIS tends to exhibit favorable ratios compared to Open S. While Open S may sometimes result in lower initial costs, its longer hospital stays increase overall costs and reduce Cost ratios. In contrast, MIS offers comparable or lower mean costs per procedure, shorter hospital stays, and favorable Cost ratios. Although MIS may require higher initial investments, its benefits of shorter hospital stays and potential cost savings improve cost-effectiveness over time.

MIS provides notable cost savings and efficiency for cholecystectomy, with no significant cost difference observed for appendectomy and ovarian cystectomy. Additionally, MIS offers societal benefits over open procedures, such as shorter stays and fewer complications, facilitating faster patient turnover, and optimizing resources, especially critical given the constraints of limited bed capacity.

This study highlights the critical role of health insurance in advancing surgical techniques and improving health outcomes across diverse population segments. With 90 % of the population covered by the CBHI scheme, Rwanda's inclusive healthcare approach ensures that even economically vulnerable individuals have access to advanced medical procedures, thus promoting equity and accessibility. The remaining 10 % is covered by RSSB for civil servants, MMI for security personnel or private insurance, underscoring the reach and impact of health insurance coverage in promoting the adoption of innovative surgical techniques across all demographics [13,16,34].

This study provides valuable insights into the application of MIS in Rwanda's two largest teaching hospitals, examining cost implications and utilization trends for common procedures. The findings reinforce the global benefits of MIS while highlighting country-specific challenges and opportunities.

MIS for cholecystectomy emerged as the most cost-effective procedure, aligning with international trends [20]. Shorter hospital stays, fewer complications, and faster recovery contributed to overall savings. In contrast, hernia repair incurred higher costs with MIS, mainly due to operative expenses and consumables, consistent with previous findings [21,22]. For appendectomy and ovarian cystectomy, cost differences between MIS and Open S were not significant, a pattern observed in studies from Nigeria and Pakistan [[24], [25], [26]].

The growing adoption of MIS in Rwanda is driven by initiatives like the MIS fellowship program supported by Belgian universities [31,32]. Cholecystectomy, frequently performed laparoscopically, serves as an entry-level procedure for training. However, hernia repair remains predominantly open, reflecting barriers such as higher equipment costs and limited training opportunities [22]. Addressing these barriers requires further investment in training, infrastructure, and policy adjustments to support MIS expansion.

Rwanda's healthcare financing plays a pivotal role in MIS adoption. With 90 % of the population covered by CBHI and additional insurance schemes for civil servants and security personnel, financial protection improves surgical accessibility [13,16,34]. However, some MIS consumables are not recorded in hospital books, as they are sourced externally, leading to potential underestimation of costs. This highlights the need for improved cost-tracking mechanisms to enhance financial planning.

While hospitalization costs depend on surgical approach and hospital stay duration, reducing inpatient stays through efficient perioperative management could optimize cost savings. Given Rwanda's goal of achieving Universal Health Coverage by 2030, a comprehensive evaluation of MIS's long-term cost-effectiveness analyzing both direct and indirect costs (recovery time, lost productivity) to evaluate the overall economic benefits of MIS, assessing infrastructure, training, and resource availability for MIS in CHUs will be essential for resource allocation and policy-making.

MIS holds promise for Rwanda's surgical landscape, offering economic and clinical advantages. Strategic investments in training, cost management, and policy integration will be crucial for scaling MIS and ensuring sustainable implementation.

## Limitations

It is important to acknowledge some of the limitations of this study. The small sample size for certain procedures, especially during the Covid-19 pandemic, when surgeries were delayed, and hospital visits avoided due to contamination risks. These factors may impact the generalizability of findings.

It was conducted as a retrospective study, which is subject to selection bias and potential missing data. The choice of laparoscopic versus open surgery may have been influenced by factors not fully accounted for in our dataset, such as surgeon preference, patient comorbidities, or intraoperative findings. A prospective study would provide a more controlled comparison.

The differences in patient characteristics between the laparoscopic and open surgery groups may have influenced cost differences. Factors such as age, comorbidities, and surgical complexity could have impacted both surgical approach selection and overall healthcare costs.

Despite these limitations, our study provides valuable insights into the economic implications of laparoscopic versus open surgery in Rwanda and highlights key considerations for healthcare policy and resource allocation.

## Conclusion

The study findings indicate that MIS procedures, notably cholecystectomies, offer cost advantages over open procedures, while Lap hernia repairs incur higher costs. However, no significant cost difference is observed for appendectomies and ovarian cystectomies. Additionally, MIS is associated with reduced complication rates and shorter hospital stays, rendering it a preferred choice for certain surgical interventions. Notably, the CBHI coverage shows higher utilization rates of MIS, highlighting benefits in accessibility and affordability.

While cost is a crucial factor in surgical decision-making, it should not overshadow other vital considerations. The benefits of MIS, such as reduced complication rates and shorter hospital stays, underscore its promising potential across various surgical interventions. Moreover, the higher utilization rates of MIS among the insured population suggest increased accessibility and affordability of advanced surgical techniques.

Addressing identified challenges and barriers within Rwanda's healthcare system is essential to capitalize on the benefits of MIS by focusing on infrastructure, training gaps, and supply chain issues. Future research efforts should focus on incorporating indirect costs, and larger sample sizes, and explore the Cost-effectiveness of MIS in a broader range of procedures, including its impact on long-term healthcare expenditures throughout the whole country. Furthermore, investigating issues related to consumables, public acceptance of MIS, Insurance coverage, and cost transparency, particularly regarding consumables and procurement policies. Finally, healthcare workforce proficiency in MIS will provide valuable insights for wider adoption within Rwanda's healthcare system, ultimately enhancing the quality of care.

## CRediT authorship contribution statement

**King Kayondo:** Writing – review & editing, Writing – original draft, Visualization, Validation, Supervision, Resources, Project administration, Methodology, Investigation, Funding acquisition, Formal analysis, Data curation, Conceptualization. **Martin Nyundo:** Supervision, Resources, Methodology, Validation. **Miguel Gasakure:** Resources, Methodology, Investigation, Formal analysis, Data curation. **Janvière Mutamuliza:** Supervision, Methodology, Investigation, Data curation. **Leon Ngeruka:** Investigation, Formal analysis. **Regis Hitimana:** Supervision, Methodology, Formal analysis. **Julien Gashegu:** Validation, Supervision, Methodology, Formal analysis. **Annie Robert:** Validation, Supervision, Methodology, Formal analysis, Conceptualization.

## Ethics approval

Approval for this study was obtained from the Rwanda National Ethical Committee (No 145/RNEC/2023), as well as CHUK and RMRTH.

## Funding sources

This research was partially funded by the Académie de Recherche et d'Enseignement Supérieur (ARES), Wallonie-Bruxelles International, through Belgian cooperation under the ARES Projet de Formation Sud (PFS) 2020 – Rwanda.

## Declaration of competing interest

Dr. King Kayondo, Dr. Martin Nyundo, Dr. Miguel Gasakure, Janvière Mutamuliza, Dr. Leon Ngeruka, Dr Regis Hitimana, Dr Prof. Julien Gashegu, and Prof. Annie Robert declare that they have no conflicts of interest or financial relationships to disclose.
